# The Reliability of Surgical Apgar Score in Predicting Immediate and Late Postoperative Morbidity and Mortality: A Narrative Review

**DOI:** 10.5041/RMMJ.10316

**Published:** 2018-01-29

**Authors:** Abhijit Nair, Aanchal Bharuka, Basanth Kumar Rayani

**Affiliations:** Department of Anesthesiology, Basavatarakam Indo-American Cancer Hospital and Research Institute, Hyderabad, Telangana, India

**Keywords:** Apgar score, morbidity, mortality, postoperative complications, risk factors, surgery

## Abstract

Surgical Apgar Score is a simple, 10-point scoring system in which a low score reliably identifies those patients at risk for adverse perioperative outcomes. Surgical techniques and anesthesia management should be directed in such a way that the Surgical Apgar Score remains higher to avoid postoperative morbidity and mortality.

## INTRODUCTION

Surgical risk scoring is important to predict postoperative outcomes, to plan admission to the intensive care unit (ICU), to prognosticate the general condition of the surgical patient, and to plan specific interventions postoperatively. A unit which is not fully equipped with multispecialty areas could plan transfer of a patient to a more specialized center based on the risk score.

Virginia Apgar, an anesthesiologist, described the 10-point scoring system, the Apgar score, in 1952 for assessing newborn babies.^1^ Scoring is done at 1 min and 5 min after birth. The score is helpful in predicting overall outcome after resuscitation of a child. Anesthesiologists and surgeons anticipate the perioperative events involved after major surgeries (laparotomies, resection/anastomosis, vascular surgery, neurosurgeries, emergency or urgent surgery) on the basis of factors like age, associated co-morbidities, surgical blood loss, and surgery duration. An otherwise uneventful intraoperative course does not predict the postoperative course in any patient. Complications occurring after surgery, especially after patient discharge, lead to increased morbidity, increased cost of treatment in the form of hospital admissions, and unwanted interventions.

## THE RISK SCORING SYSTEMS

The Physiological and Operative Severity Score for the Enumeration of Mortality and Morbidity (POSSUM), the Simplified Acute Physiology Score (SAPS), and Acute Physiology and Chronic Health Evaluation (APACHE) scoring systems have been used to predict postoperative course.

Copeland et al. initially described POSSUM in 1991, for predicting morbidity and mortality of surgical patients.^2^ In 1998, the Portsmouth modification or the P-POSSUM was described. The P-POSSUM was found to be more reliable and accurate when compared to the POSSUM described by Copeland.^3^ Twelve physiologic variables and six operative variables are used by POSSUM (Table 1). Although P-POSSUM also uses the same indices that are used for POSSUM, the equation used to calculate the score is different. All the values have to be entered, and the score is derived either by adding up or by using software. Moreover, many investigations such as hemoglobin, urea, white cell count, sodium, potassium, and electrocardiogram are required. Surgical events are also used for scoring (peritoneal soiling, multiple surgeries). There could be a lot of personal differences when certain entries are made like assessment of surgery and respiratory status. In addition, POSSUM is not applicable for trauma patients, and an overestimation of POSSUM is possible in hepatopancreatobiliary surgeries.^4^

**Table 1:** The 12 Physiologic Indices and Six Operative Indices Used for Calculating the POSSUM Score.

Physiologic Indices		Operative Indices
Age	Hemoglobin	Operative severity
Cardiac history	White cell count	Multiple surgeries
Respiratory history	Urea	Total blood loss
Pulse rate	Sodium	Peritoneal spillage
Blood pressure	Potassium	Malignancy
Glasgow coma scale	Electrocardiogram	Mode of surgery

A total of 18 indices must be entered to derive a POSSUM score. The score could be unreliable if any one index is missing.

Another score used to predict outcomes in medical and surgical patients is the Simplified Acute Physiology Score (SAPS). The SAPS II is used to score the status of patients admitted in the intensive care unit (ICU). It includes 17 variables: 12 physiologic variables, age, type of admission, and 3 disease-related variables. The SAPS II score registers the worst value of selected variables within the first 24 h after admission and can have a score between 0 and 163 points (0–116 points for physiologic variables, 0–17 points for age, and 0–30 points for previous diagnosis). Logistic regression is used to calculate the probability of death.^5^

The Acute Physiology and Chronic Health Evaluation (APACHE) II was developed using a database of North American ICU patients in 1985.^6^ It uses a score derived from 12 routine physiologic measurements taken during the first 24 h after admission, age, and previous medical issues to provide information about the severity of disease. A score from 0 to 71 is calculated based on these measurements. A higher score signifies a more severe disease with greater risk of death. The APACHE II has been used to prognosticate acutely ill patients and has helped researchers to compare the efficacy of various forms of treatment modalities. However, APACHE II led to an overestimation of mortality as physiologic variables used were dynamic and kept changing during treatment. Later APACHE III was introduced with two new variables: patient origin and the lead-time bias. Here, the score varied between 0 and 299 points.^7^ Later, APACHE IV was introduced in which another five variables were added: mechanical ventilation, thrombolysis, impact of sedation on Glasgow Coma Score (GCS), re-scaled GCS, and PaO2/FiO2 (arterial oxygen tension and fractional concentration of inspired oxygen) ratio.^8^

The SAPS and APACHE were found to be more reliable in predicting severity of condition and outcomes in medical patients when compared to surgical patients.^9^ Rapsang et al. have described nine routinely used scoring systems for predicting the morbidity and mortality of patients admitted in the ICU. The authors felt that selecting an inappropriate scoring methodology could lead to a significant waste of time, unwanted investigations, increased cost, and unwarranted extrapolations.^10^ Anesthesiologists use the American Society of Anesthesiologists–Physical Status (ASA-PS) classification for describing patients based on co-morbidities, functional status, and emergency or elective surgery. The ASA-PS was not designed to predict the mortality of a surgical patient. The ASA classification, with a positive predictive value of 57% for complications and a negative predictive value of 80%, is not considered reliable for predicting the 30-day postoperative course accurately.^11^

## THE SURGICAL APGAR SCORE

Gawande et al. described the Surgical Apgar Score (SAS) in 2007.^12^ The score was derived from a retrospective analysis of 303 patients who underwent colectomy at Brigham and Women’s Hospital, Boston, MA. This 10-point score is based on the patient’s surgical blood loss, the lowest intraoperative heart rate, and lowest recorded mean arterial pressure. The authors observed that as the score increased, outcomes improved at the end of 30 days. Many papers (discussed later on in this article) were subsequently published that interpreted prospective and retrospective data and concluded that SAS could accurately predict morbidity and complications in several surgical subspecialties. The SAS uses a 10-point scoring system that has been used to accurately predict early and 30-day postoperative complications in all major surgeries in the last decade. The 10-point SAS is shown in Table 2.

**Table 2:** The 10-point Surgical Apgar Score.

Parameters	0 Points*	1 Point	2 Points	3 Points	4 Points
Estimated blood loss (mL)	>1000	601–1000	101–600	≤100	–
Lowest mean arterial pressure (mmHg)	<40	40–54	55–69	≥70	–
Lowest heart rate (beats/min)	>85	76–85	66–75	56–65	≤55

* Occurrence of pathological bradyarrhythmia (including sinus arrest, atrioventricular block of dissociation, junctional or ventricular escape rhythms) and asystole also receives 0 points for lowest heart rate.

Reprinted from Gawande et al.^12^, ©2007, with permission from the American College of Surgeons.

## APPLICATION OF SAS BY OTHER RESEARCHERS IN OTHER SURGERIES

The correlation of SAS with perioperative morbidity and complications was different when used in different subspecialties by different researchers. However, when the patient’s ASA-PS classification was adjusted using relevant software, SAS scores remained associated with death and complications in several subspecialties. We searched PubMed, Scopus, Embase, and Google Scholar databases with the keywords “Surgical Apgar Score,” “Postoperative complications,” “Surgery,” “Morbidity,” and “Mortality” and identified 25 retrospective studies and 11 prospective studies that used SAS as a prognosticator tool to correlate with early and late postoperative complications (up to 30 days). The details of the retrospective and prospective data, type of surgical patients reviewed, total number of patients reviewed, and the reliability of SAS in predicting postoperative events are presented in Tables 3 and 4.^13^–^49^ Most of the published papers that have investigated the efficacy of SAS are based on retrospective data collected from electronic hospital records; the SAS was calculated from the records. The authors used univariate and multivariate analyses to assess factors associated with major postoperative complications. Data collected from the National Surgical Quality Improvement Program underwent logistic regression using 27 preoperative variables as predictors; the outcome was determined by using the incidence of major postoperative complications to generate a multivariable preoperative risk prediction model.

**Table 3:** All Retrospective Studies Using SAS Scores for Various Surgeries to Predict Immediate and Delayed Postoperative Complications (30 days).

Surgery Type (# of Patients) Ref.	Prognostic Value (Y/N)	Remarks
Knee arthroplasty (3,511)^13^	No	The authors felt SAS was insufficient for prognostication
Colectomy (795)^14^	Yes	SAS predicted inpatient as well as late post-discharge complications
General/vascular surgery (4,119)^15^	Yes	
Major intra-abdominal surgeries (8,501)^16^	Yes	
Esophagectomy (189)^17^	Yes	SAS predicted major morbidity associated with longer hospital stay
Esophagectomy (168)^18^	Yes	
Ivor Lewis (234)^19^	No	SAS could not predict adverse outcomes
Esophagectomy (399)^20^	Yes	
Gastrectomy (328)^21^	No	Original SAS not found useful; modified SAS was helpful in predicting complications
Hysterectomy for malignancy (632)^22^	No	SAS uncorrelated with postoperative events
Pancreatoduodenectomy (2012)^23^	Yes	
Intracranial and spine neurosurgery (918)^24^	Yes	
Surgery for spinal metastasis (97)^25^	No	SAS an insignificant predictor of major perioperative complications following spinal metastasis surgery; preoperative functional status and age were stronger predictors
Lower extremity amputations (228)^26^	Yes	Predicted potential development of complications
Wide surgical subspecialties (123,864)^27^	Yes	
Intracranial meningioma excision (999)^28^	Yes	SAS predicted early and late complications
Pancreatoduodenectomy (103)^29^	Yes	SAS was a significant independent risk factor for overall and recurrence-free survival
Radical prostatectomy (994)^30^	Yes	
Lumbar spine fusion (199)^31^	Yes	
Gastrectomy (191)^32^	Yes	SAS predicted survival after surgery
Major intra-abdominal surgery (629)^33^	Yes	SAS predicted survival after surgery
Kidney transplant (204)^34^	Yes	SAS correlated with ICU stay and overall cost of treatment
Microvascular head and neck reconstruction (154)^35^	No	SAS uncorrelated with postoperative complications
Surgery for traumatic hip fractures (43)^36^	Yes	
Pancreatic resection (143)^37^	Yes	SAS along with hypoalbuminemia and blood transfusion correlated well with hospital stay and complications
Major gastrointestinal surgeries (1,833)^38^	Yes	The authors modified SAS by including intraoperative blood transfusion and assigned zero estimated blood loss (EBL) score to patients who received transfusion; they concluded that intraoperative transfusion improved risk stratification of SAS

**Table 4:** Prospective Studies Using SAS Scores for Various Surgeries to Predict Immediate and Delayed Postoperative Complications (30 days).

Surgery Type (# of Patients) Ref.	Prognostic Value (Y/N/Insignificant)	Remarks
General/vascular surgery (143)^39^	Insignificant	Suggested conducting randomized control trial
Spine (268)^40^	Yes	
General orthopedic (723)^41^	No	SAS did not predict 30-day major complications after general orthopedic surgery
Radical cystectomy (155)^42^	Yes	
General surgery (2,125)^43^	Yes	
Laparotomy (218)^44^	Yes	
Non-cardiac surgeries (5,909)^45^	Yes	
General and vascular surgeries (224)^46^	Yes	
General, vascular, and orthopedic surgeries (223)^47^	Yes	SAS uncorrelated with orthopedic patients who had major events
Renal mass excision (886)^48^	Yes	
High-risk intra-abdominal surgeries (355)^49^	Yes	SAS was significantly predictive but weakly discriminative for adverse events

Twenty out of 25 retrospective studies concluded that SAS correlated with adverse postoperative events. The SAS could not predict unfavorable events in patients who underwent knee arthroplasties (Wuerz et al.^13^), hysterectomy for malignancy (Clark et al.^22^), Ivor Lewis esophagectomies (Strøyer et al.^19^), spine surgery for metastasis (Lau et al.^25^), gastrectomy (Miki et al.^21^), and microvascular head and neck reconstruction (Ettinger et al.^35^). The authors felt that preoperative functional status and age were stronger predictors than SAS. Out of the 11 prospective studies, one study that analyzed patients undergoing orthopedic surgeries suggested that SAS could not predict adverse surgical outcomes. Haddow et al.^39^ analyzed 143 general and vascular surgical patients, and suggested conducting a randomized control trial due to the few cases, which provided inconclusive data. Thorn et al.^47^ found that general and vascular surgery patients with a lower SAS correlated well with postoperative outcomes. However, there was poor correlation in orthopedic patients. The initial hypotension that occurs after administering a spinal anesthetic may explain the poor SAS correlation of postoperative events in these patients.

Hypotension usually improves with crystalloid boluses or a few doses of vasopressors. House et al.^50^ retrospectively analyzed data from 2007–2012 and found that a low SAS score was due to increased cardiac troponin levels after non-cardiac surgery. Out of 46,799 patients, 209 (0.4%) had increased troponins and 192 (0.4%) patients experienced myocardial infarction following non-cardiac surgeries.^50^ Jering et al.^51^ used the Area Under the Receiver Operating Characteristic Curve (AUROC) and suggested that combining ASA physical classification with continuously measured SAS was better in predicting major postoperative complications than using ASA physical status and SAS alone.^51^

## DISCUSSION

An ideal surgical risk scoring system should be simple; require minimal calculation, data and variables; be reasonably accurate; and must be objective, economical, and suitable for all situations (elective/emergency surgeries and valid in all specialties). The SAS is a simple way to predict complications during the postoperative period. It is a simple and inexpensive scoring system that can reliably predict serious postoperative consequences relying on only three variables. Patients undergoing major thoracic, abdominal, and vascular surgeries with significant co-morbidities are expected to have adverse perioperative outcomes. In spite of this, the SAS was able to predict either alone, or in combination with associated risk factors, the occurrence of life-threatening events in the postoperative period. A patient with a low intraoperative SAS should be considered at risk and monitored meticulously. A patient with a low SAS should be monitored for an extended period in the ICU.

The SAS does not appear to correlate well with surgeries performed under regional anesthesia (e.g. arthroplasties, as shown by Wuerz et al.^13^ and Thorn et al.^47^). Well-designed prospective studies in the future could provide better insight into the reason behind the lack of correlation between regional anesthesia and SAS. The score could also help surgeons to improve or change their practice. This might include, for example, preventing surgical bleeds by meticulous use of electrocautery, identifying and ligating possible bleeders, and/or using a tourniquet whenever possible; giving time for bleeding to maintain mean arterial pressure (MAP); and avoiding events that lead to severe bradycardia by using slow insufflations after port insertion, maintaining normal intra-abdominal pressure, and avoiding forceful omental/peritoneal handling.

Hyder et al.^52^ investigated the effect on the SAS of different sampling methods for extracting vital signs data. In the study that involved more than 3,000 patients, they found that larger SAS sampling intervals resulted in better model discrimination and improved reclassification. The authors had a large sample size, studied a variety of non-cardiac surgeries, and had a detailed classification of preoperative and postoperative morbidity. Optimized algorithms and larger sampling intervals of required parameters are needed to use SAS to predict patients at risk for adverse postoperative events. Smaller sampling intervals could lead to inadequate data, leading the investigator to find SAS unsatisfactory in predicting adverse outcomes.

## CONCLUSION

The SAS is a simple 10-point scoring system that can be easily calculated and entered in case records at the end of surgery. Unlike other scoring systems, SAS does not require biochemical investigations, clinical assessment, acute or chronic disease classification, or depend on the timing of the surgery (elective, urgent, emergency). The patient with a low SAS could experience adverse perioperative life-threatening events during the first 30 days of the postoperative period. Using SAS helps to identify patients at risk, and contributes to post-procedural auditing with evidence-based methodologies to help achieve the highest possible SAS in a surgical patient. In addition, SAS along with ASA-PS and larger sampling intervals could help to identify patients in need of monitoring and vigilant follow-up during the postoperative period.

## Abbreviations

APACHE: Acute Physiology and Chronic Health Evaluation
ASA-PS: American Society of Anesthesiologists–Physical Status
GCS: Glasgow Coma Score
ICU: intensive care unit
POSSUM: Physiological and Operative Severity Score for the Enumeration of Mortality and Morbidity
P-POSSUM: Portsmouth modification POSSUM
SAPS: Simplified Acute Physiology Score
SAS: Surgical Apgar Score
